# Macroglossia Associated With Clobazam Administration: A Case Report and Literature Review

**DOI:** 10.3389/fneur.2022.900763

**Published:** 2022-05-09

**Authors:** Jeff F. Zhang, Kevin Nickerson, Ravi Piryani, Osman Farooq, Anil K. Swayampakula

**Affiliations:** ^1^Jacobs School of Medicine and Biomedical Sciences, University at Buffalo, Buffalo, NY, United States; ^2^Division of Critical Care Medicine, Department of Pediatrics, John R. Oishei Children's Hospital, Buffalo, NY, United States; ^3^Division of Pediatric Neurology, John R. Oishei Children's Hospital, Buffalo, NY, United States

**Keywords:** antiseizure drug, Clobazam, epilepsy, macroglossia, pediatric neurology

## Abstract

Clobazam is a benzodiazepine derivative used as an antiepileptic agent for the treatment of focal and generalized seizures and drug-resistant epilepsy associated with Lennox-Gastaut Syndrome. While somnolence and mood-related side effects are commonly observed, acute macroglossia following initiation of Clobazam therapy has not been previously reported in the medical literature. In this case report, we present a female pediatric patient who developed significant tongue swelling with protrusion beyond the oral cavity after initiation of Clobazam for treatment-resistant epilepsy. Symptoms were unresponsive to antihistamines and steroids but resolved gradually in the days following discontinuation of Clobazam with no lingering sequelae.

## Introduction

Macroglossia is defined as a painless enlargement of the tongue that presents either congenitally in association with a wide variety of inherited disorders (e.g., Down Syndrome, Hunter and Hurler Syndromes, Beckwith-Wiedemann Syndrome) or acquired as a result of inflammation, amyloidosis, and endocrine and metabolic disorders. In rare cases, severe tongue swelling causing upper airway obstruction presents as a medical emergency requiring reduction glossectomy, which is indicated in an estimated maximum of 10% of cases of macroglossia (1). Frequently, acquired macroglossia resolves spontaneously with treatment of the underlying cause, and corticosteroids have also shown effectiveness in reducing glossal edema associated with trauma and mechanical injury (2).

Macroglossia secondary to angioedema is commonly observed in the setting of drug hypersensitivity reactions, in which drug-antigen presentation causes crosslinking of IgE immunoglobulins on the surface of mast cells with subsequent degranulation of intracellular contents. The release of vasoactive substances such as histamine and bradykinin leads to increases in the permeability of submucosal and subcutaneous capillaries, resulting in fluid shifts into the interstitial environment that cause localized tissue swelling. The onset of glossal swelling following the administration of Clobazam therapy for refractory epilepsy has not been previously reported in the literature and the pathophysiology by which these symptoms occur is currently unknown. In this case report, we present a pediatric patient who developed significant tongue swelling with protrusion beyond the oral cavity in the days after initiating Clobazam therapy for treatment-resistant epilepsy and whose symptoms resolved gradually following discontinuation of the drug.

## Case Presentation

**Figure 1:**
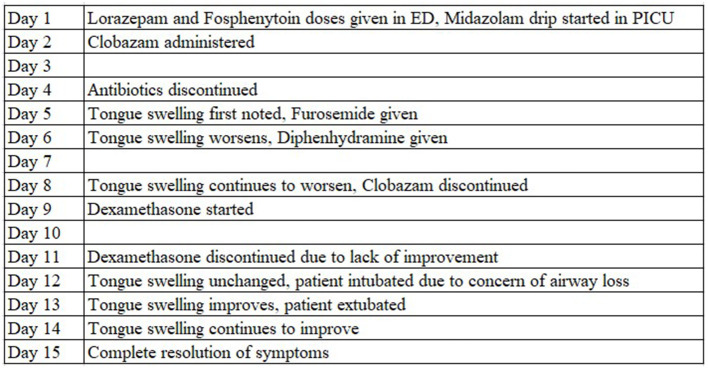
Timeline of patient hospitalization events.

A 17-year-old Caucasian female, born at 34 weeks *via* Cesarean section, presented to the emergency department (ED) with status epilepticus. Her past medical history was significant for unbalanced chromosome 6 and 21 translocation, gross developmental delay, polymicrogyria, low-lying cerebellar tonsils, arachnoid cyst, micrognathia, scoliosis, and multifocal drug-resistant epilepsy since age 5 (managed on Levetiracetam, Lacosamide, and rectal Diazepam as needed). At baseline, the patient experiences four seizures per day lasting between 30 and 60 s and the parents report the patient maintains good compliance with her antiseizure drug regimen. In the 24 h prior to admission, the father estimated that she had ~80 breakthrough seizures with episodes of head deviation and whole body stiffening and shaking each lasting between 30 and 120 s in duration. No significant improvement was observed after giving extra doses of Levetiracetam and Lacosamide. The family denied any recent history of fever, upper respiratory infection, nausea, vomiting, urinary symptoms, trauma, or travel. Previous attempts to control the patient's seizures using Topiramate, Sodium Valproate, Oxcarbazepine, Zonisamide, and Lamotrigine were ineffective. Figure 1 represents the timing of events in the patient's hospital course by day of admission.

**Figure 2:**
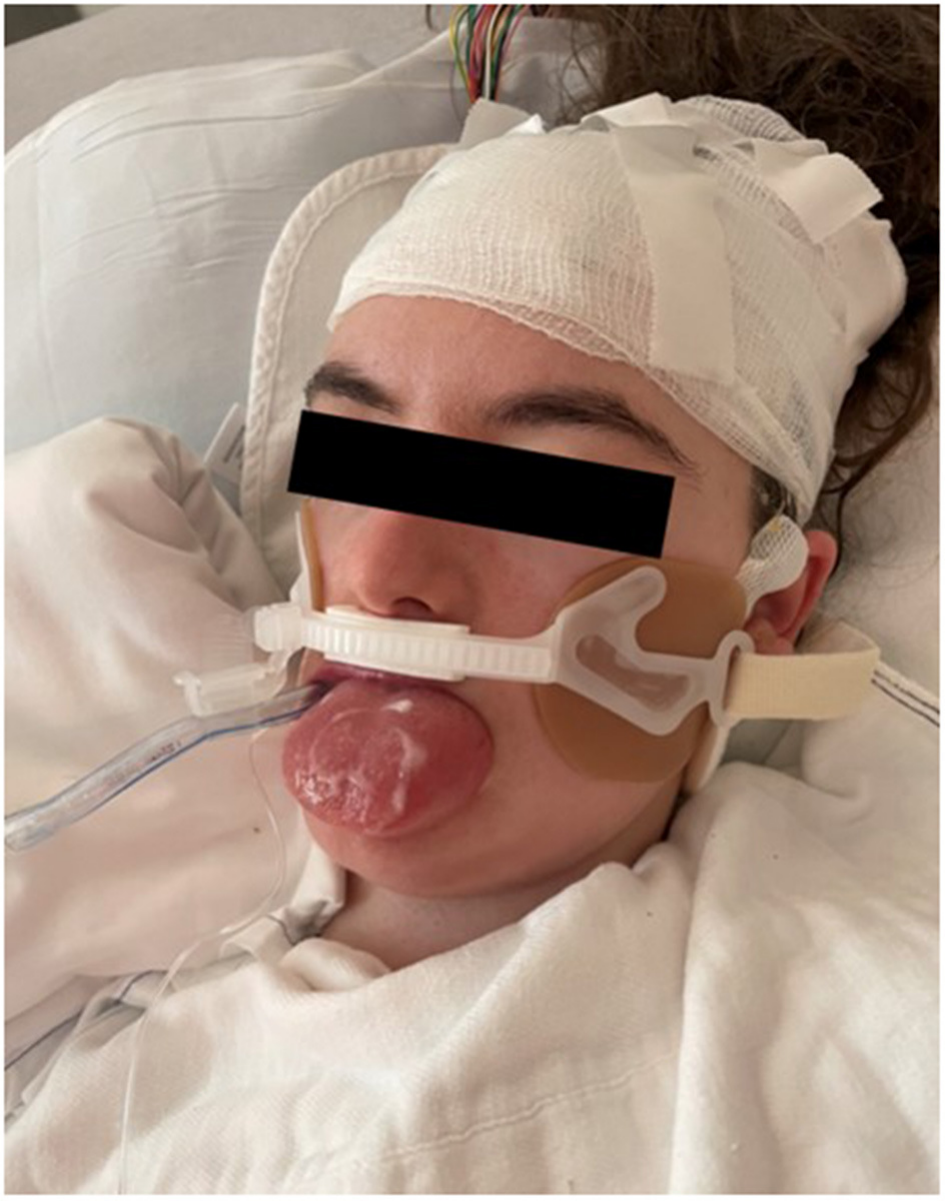
Patient with notable tongue enlargement and protrusion from oral cavity.

Lab results showed leukocytosis (WBC, 15.1 × 10^9^/L) with neutrophils 59.9% and lymphocytes 32.0%, and reactive thrombophilia (482 × 10^9^/L). Comprehensive metabolic panel was significant only for anion gap elevation (16 mmol/L) and low bicarbonate (15 mmol/L) which resolved over the following days. Ceftriaxone was started for empiric coverage; initial blood cultures grew Staphylococcal species and Vancomycin was added. The patient's seizures were terminated by Lorazepam and Fosphenytoin loading in the ED. She was continued on her home antiseizure medications and continuous video electroencephalography (EEG) was started. The patient was stabilized in the ED and transferred to the pediatric floor. Overnight, the patient began to have seizures again and EEG detected epileptogenic sharp waves over the left hemisphere. Due to continued seizures, the patient was subsequently transferred to the pediatric intensive care unit (PICU), started on a Midazolam drip, and required endotracheal intubation for airway protection. Clobazam 5 mg BID was initiated on Day 2 of hospital admission for further seizure control. Head CT and brain MRI were negative for acute intracranial pathology. Antibiotics were discontinued on Day 4 following negative blood, urine, and cerebrospinal fluid cultures, and the patient's seizures were reported to be adequately controlled at this time. On Day 5 of PICU admission, the patient was noted to have mild tongue swelling; this was initially thought to be secondary to fluid overload and the patient was started on Furosemide given her overall volume status. While her volume status notably improved, her tongue swelling continued to worsen, with inability to manually retract the tongue and protrusion beyond the oral cavity over the following day (Figure 2). No signs of infection or trauma were noted within the oral cavity on inspection. Trial of Diphenhydramine did not improve the tongue swelling. Given that Clobazam was the only new medication that she had not previously received, it was suspected to be the offending agent and replaced with Clonazepam on Day 8 of hospitalization. No recurrence of seizures were noted and EEG was discontinued. On the following day, a steroid course with Dexamethasone was initiated for the patient's macroglossia. Serum complement panel demonstrated normal C4 levels (39.6 mg/dL) and elevated C1 esterase inhibitor (53 mg/dL). Further inquiry into family history was also negative for angioedema. On Day 12, following 72 h of steroid administration, the patient's tongue was noted to still be edematous and protruding. Intubation and mechanical ventilation had been maintained due to concern of airway loss from macroglossia. On Day 13, she started to show improvement with tongue swelling and was successfully extubated with no complications. Complete resolution of macroglossia was observed on Day 15 of hospitalization, seven days following the discontinuation of Clobazam.

## Discussion

Clobazam is a benzodiazepine antiepileptic agent used to treat a range of focal and generalized seizure disorders, as well as drug-resistant seizures associated with syndromes such as Lennox-Gastaut Syndrome and Dravet Syndrome. Its mechanism of action is through its binding to γ-aminobutyric acid-A (GABA-A) receptors and potentiation of GABA action by increasing the channel conduction of chloride ions in response to ligand binding, thereby increasing the seizure threshold (3). Clobazam differs from typical benzodiazepines due to placement of its two nitrogen atoms at the 1st and 5th positions of the diazepine ring resulting in partial agonism of the GABA-A receptor, rather than the 1st and 4th ring positions of other benzodiazepines which act as full agonists (3). This structural variation confers an improved side effect profile and also increases its anxiolytic and antiepileptic properties due to improved binding affinity for the GABA-A α2β3γ2 subtype (4). Common side effects noted in up to 80% of patients during Phase III drug trials included somnolence, pyrexia, lethargy, drooling, and constipation (5). While side effects are generally mild, increased severity has been observed in patients prescribed Clobazam along with cannabinoid agents such as Epidiolex for the treatment of refractory epilepsy (6). Serological studies show cannabidiol use in combination with Clobazam was correlated with a five-fold increase in concentration of N-desmethylclobazam, the active metabolite of Clobazam, due to shared metabolism by the hepatic cytochrome P450 enzyme CYP2C19 (7). Patients with “poor metabolizer” phenotypes due to high rates of polymorphism in the CYP2C19 gene have also been noted to be at a higher risk of adverse drug effects (8). However, the patient described in our case report did not have a history of cannabinoid use and CYP2C19 genotyping had not been conducted.

**Figure 3:**
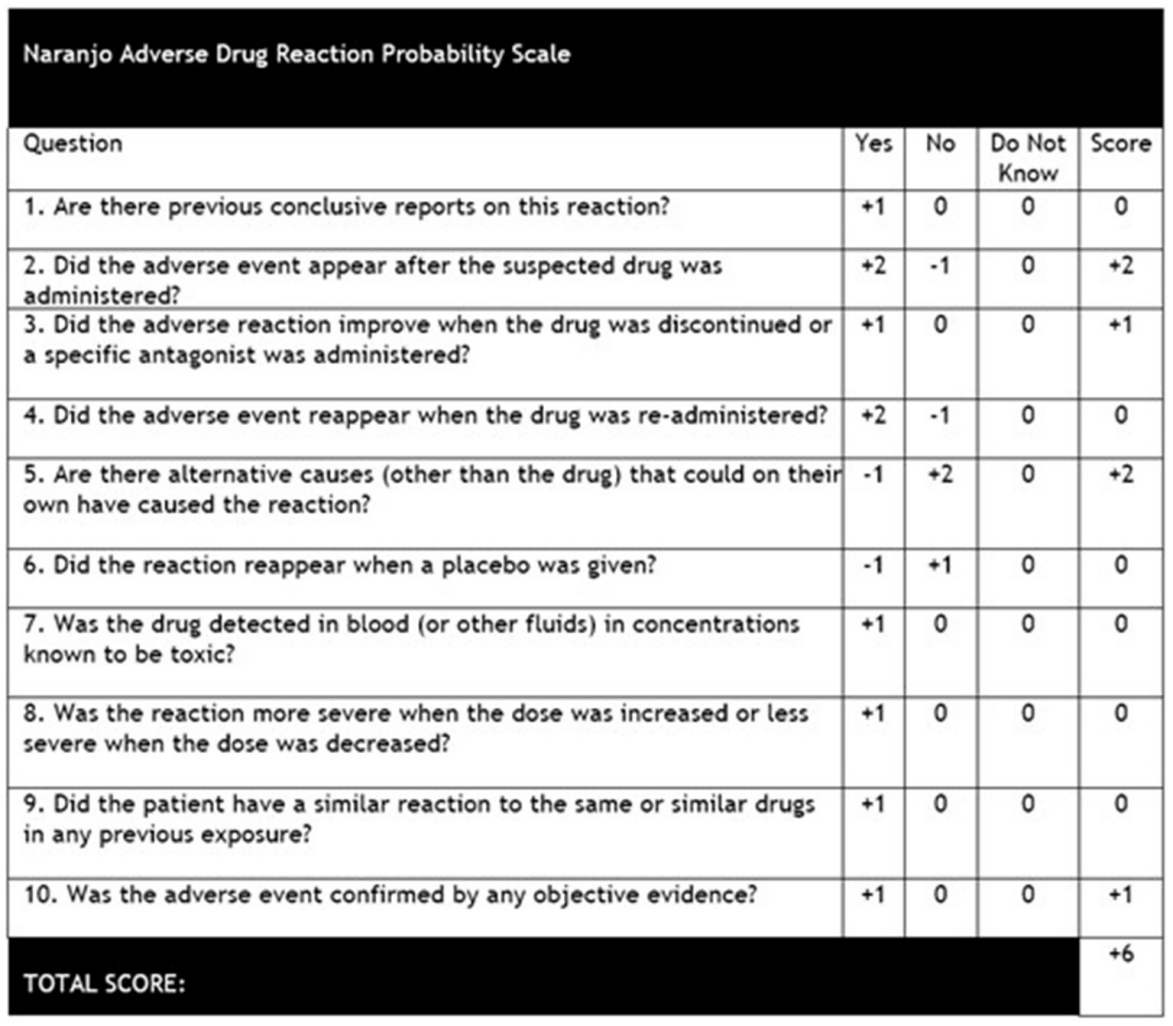
Naranjo adverse drug reaction probability scale (9). Score was calculated as “+6” due to the patient's adverse event appearing following administration of Clobazam, improvement of symptoms following discontinuation of the drug, other causes for the patient's tongue edema ruled out, and the results of the patient's presentation and work-up confirmed with neurology, immunology, and otolaryngology consults.

Our report presents the first published case of acute tongue enlargement in the context of naïve Clobazam exposure. While the patient had also been treated with antibiotics, Lorazepam, Fosphenytoin, and Midazolam during her inpatient course, the patient's previous exposure to these medications, lack of rash or pruritus, and minimal response to Diphenhydramine and corticosteroid therapy likely rule out allergic or inflammatory etiologies. In addition, our findings of elevated C1-esterase inhibitor levels, normal C4 complement levels, and negative family history for angioedema accounted for any possible pathologies related to bradykinin-mediated angioedema. Improvements in macroglossia observed while the patient was still intubated and lack of similar symptoms following previous intubations also make a traumatic cause for the patient's acute tongue swelling less likely. However, calculation of the Naranjo adverse drug reaction probability scale (9) for our patient (represented in Figure 3) yielded a score of “+6,” which determined a “Probable” drug-related cause for the patient's macroglossia, and it had been noted that Clobazam was the only new medication that the patient had been exposed to during this hospital stay. While isolated tongue swelling has not previously been described in the literature following Clobazam administration, two reports have presented four adult patients (10) and one pediatric patient (11) who developed distal extremity edema associated with chronic Clobazam use (within 1–3 months of therapy) that spontaneously resolved within 2–6 weeks following discontinuation of the drug. In both cases, the pathophysiology was speculated to be due to the disruption of autonomic signaling in the myocardium and peripheral vasculature due to benzodiazepine-mediated increases in chloride channel conduction (12). However, no reports of macroglossia or extremity edema have been reported in the acute setting in the days following initiation of Clobazam therapy.

Notably, acute tongue swelling has been reported in patients receiving barbiturates for sedation. Darshan et al. reported a patient who developed severe tongue swelling with protrusion after placement into a drug-induced coma using Pentobarbital (13). The patient was weaned off Pentobarbital with improvement of his tongue swelling over the following week; however, later in his hospital course, he was administered Pentobarbital again and three days later developed similar symptoms of macroglossia, which again resolved in a few days following discontinuation of Pentobarbital (13). In another report, Parthvi et al. reported a patient who was administered Phenobarbital for agitation related to alcohol withdrawal and also developed isolated tongue swelling that was ruled to not be related to anaphylaxis or angioedema (14). In a similar mechanism of action to benzodiazepines, barbiturates act as positive allosteric modulators of GABA-A receptors by binding to a distinct site from benzodiazepines and increasing GABA-mediated activity. Phenobarbital and Pentobarbital have also been found to be mainly metabolized by hepatic CYP2C19 (15), and patients with genetic polymorphisms resulting in decreased enzymatic activity have been noted to be at higher risk of developing side effects related to barbiturate intake (16). We would hypothesize that in patients with a poor metabolizer phenotype characterized by reduced CYP2C19 activity, elevated drug metabolite levels disrupt autonomic vasoconstriction in the tongue (a highly neurovascular structure) and produce the characteristic macroglossia observed in these patients, though further serological and genotyping studies are required to draw definitive conclusions in this susceptible patient population.

Unresolved macroglossia poses a significant risk for unsuccessful extubation, resulting in obstructive symptoms post-extubation or possible cardiac arrest due to an inability to pass an oral or nasotracheal tube and necessitating emergency tracheostomy. Fortunately, as in the case of our patient, drug-related symptoms of macroglossia have been observed to resolve quickly and spontaneously following discontinuation of the offending agent without any lingering sequelae. In this case report, we hope to increase awareness among clinicians for this possible side effect of Clobazam therapy in order to avoid unnecessary testing and treatment which may prolong a patient's length of stay.

## Conclusions

Macroglossia is a previously unreported side effect associated with initiation of Clobazam therapy for drug-resistant epilepsy. Early recognition of this symptom and discontinuing the offending agent may to lead to spontaneous resolution of macroglossia and can reduce hospital length of stay.

## Data Availability Statement

The original contributions presented in the study are included in the article/supplementary material, further inquiries can be directed to the corresponding author/s.

## Ethics Statement

Written informed consent was obtained from the individual(s), and minor(s)' legal guardian/next of kin, for the publication of any potentially identifiable images or data included in this article.

## Author Contributions

JZ: drafting the manuscript, editing the manuscript, approving the manuscript, and accountable for manuscript integrity. KN and RP: editing the manuscript and approving the manuscript. OF and AS: project conception, editing the manuscript, and approving the manuscript. All authors contributed to the article and approved the submitted version.

## Conflict of Interest

The authors declare that the research was conducted in the absence of any commercial or financial relationships that could be construed as a potential conflict of interest.

## Publisher's Note

All claims expressed in this article are solely those of the authors and do not necessarily represent those of their affiliated organizations, or those of the publisher, the editors and the reviewers. Any product that may be evaluated in this article, or claim that may be made by its manufacturer, is not guaranteed or endorsed by the publisher.
